# Data on atomic structures of precipitates in an Al-Mg-Cu alloy studied by high resolution transmission electron microscopy and first-principles calculations

**DOI:** 10.1016/j.dib.2021.106748

**Published:** 2021-01-15

**Authors:** Xuanliang Chen, Calin D. Marioara, Sigmund J. Andersen, Jesper Friis, Adrian Lervik, Randi Holmestad, Equo Kobayashi

**Affiliations:** a Department of Materials Science and Engineering, Tokyo Institute of Technology, 2-12-1-S8-18, Ookayama, Meguro-ku, Tokyo, 152-8552, Japan; b Materials and Nanotechnology, SINTEF Industry, Høgskoleringen 5, N-7465, Trondheim, Norway; c Department of Physics, Norwegian University of Science and Technology (NTNU), Høgskoleringen 5, N-7491, Trondheim, Norway

**Keywords:** Aluminum alloys, Precipitation, TEM, DFT, Atomic models

## Abstract

The dataset refers to the research article “Precipitation processes and structural evolutions of various GPB zones and two types of S phases in a cold-rolled Al-Mg-Cu alloy” [1]. Transmission electron microscopy (TEM) and density functional theory (DFT) were used to investigate precipitates in an Al-Cu-Mg alloy aged at 443 K for various times. High-angle annular dark-field scanning TEM (HAADF-STEM) images in <100> Al orientations were analyzed. Characteristic contrast and symmetries of columns [2] yielded atoms and positions, used to build precipitate models which could be refined and compared with solid solution reference energies. A calculation cell is an Al supercell compatible with symmetry and morphology of a precipitate, which is fully or partly surrounded by Al, allowing periodicity continuation via neighbor cells. The given crystallographic data include two S-phase variants and Guinier–Preston–Bagaryatsky (GPB) zones, of which the “GPBX” is new.

## Specifications Table

**Table:** 

Subject	Materials Science
Specific subject area	Precipitates in Al-Mg-Cu alloys
Type of data	1. Image (HAADF-STEM images) 2. Table (Crystal structure data of precipitate models and refinements)
How data were acquired	1. In a transmission electron microscope (double Cs corrected JEOL ARM 200F). 2. Models manually extracted from HAADF images. Images were contrast enhanced using Gatan Microscopy Suite (GMS) and the freely available computer software package ImageJ. Atoms were identified using rules of columns [2]. Interatomic distances and symmetry operations were checked, and space group determined using VESTA [3]. The models were input and refined using the Vienna ab-initio simulation package (VASP).
Data format	Raw Analyzed Filtered
Parameters for data collection	1. Precipitates were imaged using a HAADF in STEM mode, in <100>_Al_ orientations of regions of thickness typically less than 5 nm. 2. The models were manually extracted from the images, calculation cells tried out, input in VESTA, where after suitable cells were determined, and initial atomic coordinates calibrated using the assumed Al-Al inter-column distance 2.025Å. The calculations were performed at zero Kelvin using the projector augmented wave method within the PBE (Perdew–Burke–Ernzerhof) generalized gradient approximation [4]. The plane-wave energy cut-off was 400 eV and a Monkhorst-Pack gamma-centered k-point mesh was used, with maximal k-point distances of 0.18 Å^−1^ in each direction [5]. The electronic accuracy for self-consistent loops was set at 10^−6^ eV and the atomic positions were relaxed to a maximal force of 0.001 eV/Å between atoms using first-order Methfessel-Paxton for smearing of partial occupation and a smearing factor (SIGMA) of 0.2.
Description of data collection	1. HAADF-STEM images were taken in a double Cs corrected JEOL ARM 200F operated at 200 kV 2. Structural data were calculated from atomistic simulations using VASP.
Data source location	1. TEM data were collected at Dept. of Physics, Norwegian University of Science and Technology (NTNU), Høgskoleringen 5, N-7491, Trondheim, Norway. 2. DFT calculation data were collected at SINTEF Industry, Høgskoleringen 5, N-7465, Trondheim, Norway.
Data accessibility	With the article.
Related research article	X. Chen, C.D. Marioara, S.J. Andersen, J. Friis, A. Lervik, R. Holmestad, E. Kobayashi, Precipitation processes and structural evolutions of various GPB zones and two types of S phases in a cold-rolled Al-Cu-Mg alloy, Materials and Design. https://doi.org/10.1016/j.matdes.2020.109425.

## Value of the Data

• The atomic resolution HAADF-STEM data reported here clearly show the microstructures of Al-Mg-Cu alloys under different conditions, especially the atomic structure of various GPB zones and S phases formed during the aging treatment. The atomic models of precipitates constructed in this article give us a more intuitive impression of the precipitated phase structures. DFT calculation helps to understand the precipitation process.
• Researchers who investigate the precipitation process or precipitates structures in Al-Mg-Cu alloys can benefit from the data.
• The data can be used as a reference for other researchers to obtain specific precipitates and to optimize the precipitation heat treatment conditions.

## 1 Data Description

### 1.1 HAADF-STEM images

**Fig. 1:**
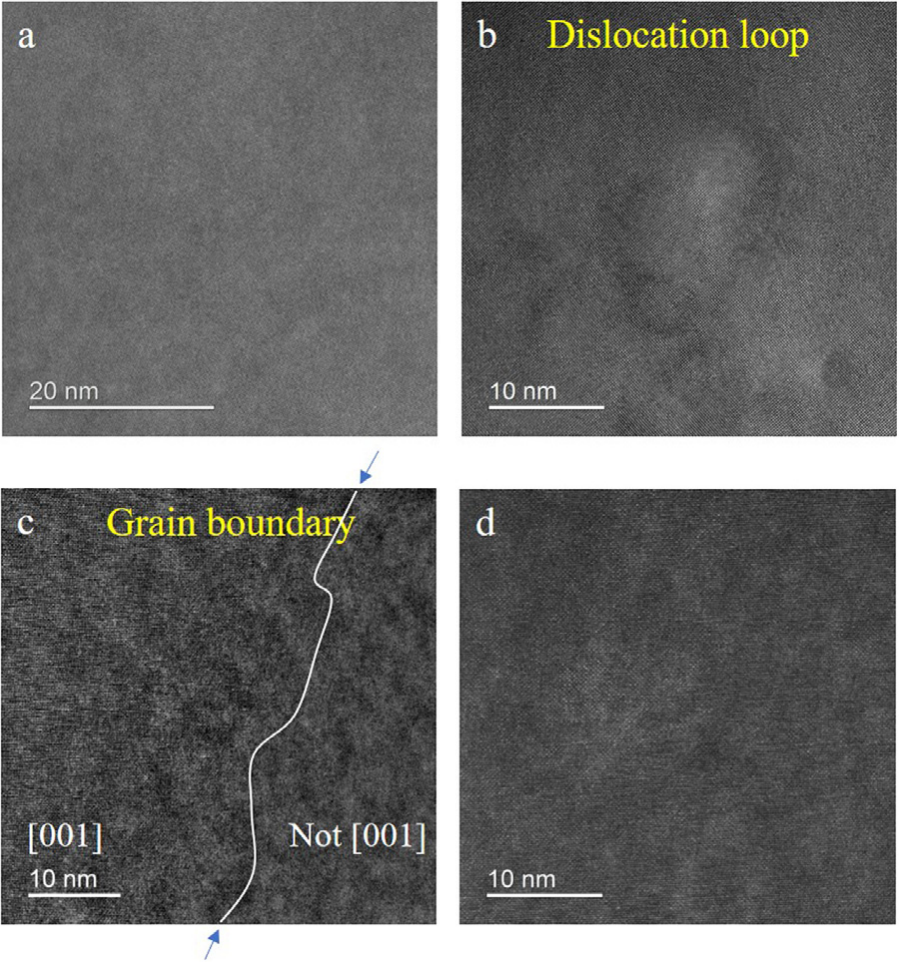
HAADF-STEM images of the as-quenched (A.Q.) and as-rolled (A.R.) samples, showing no precipitate has been formed. (a) The Al matrix in the A.Q. sample. (b) Example of quenched in dislocation loop in the A.Q. sample. (c) (FFT filtered image) Two grains in the A.R. sample, separated by blue arrowed grain boundary. The left side grain is orientated in a <001>_Al_ direction, while the right side is different since no clear atomic arrangement cannot be found. (d) The Al matrix in the A.R. sample.

**Fig. 2:**
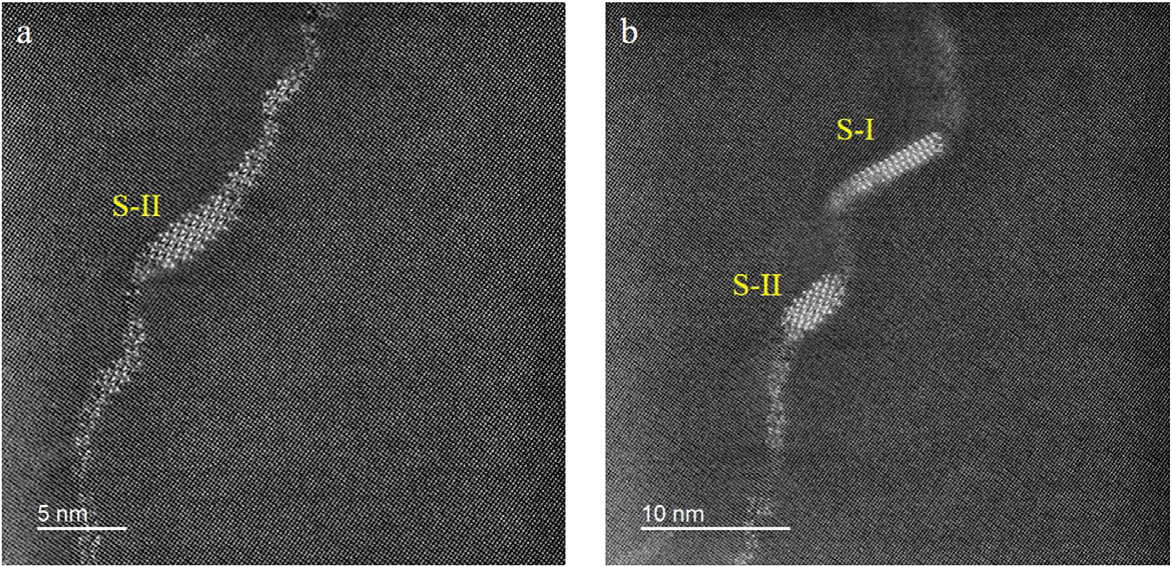
FFT filtered HAADF-STEM images in the A.Q. sample aged at 443 K for 8 hours. (a) The S-II phase, (b) Both the S-I and S-II phase were found on dislocations.

**Fig. 3:**
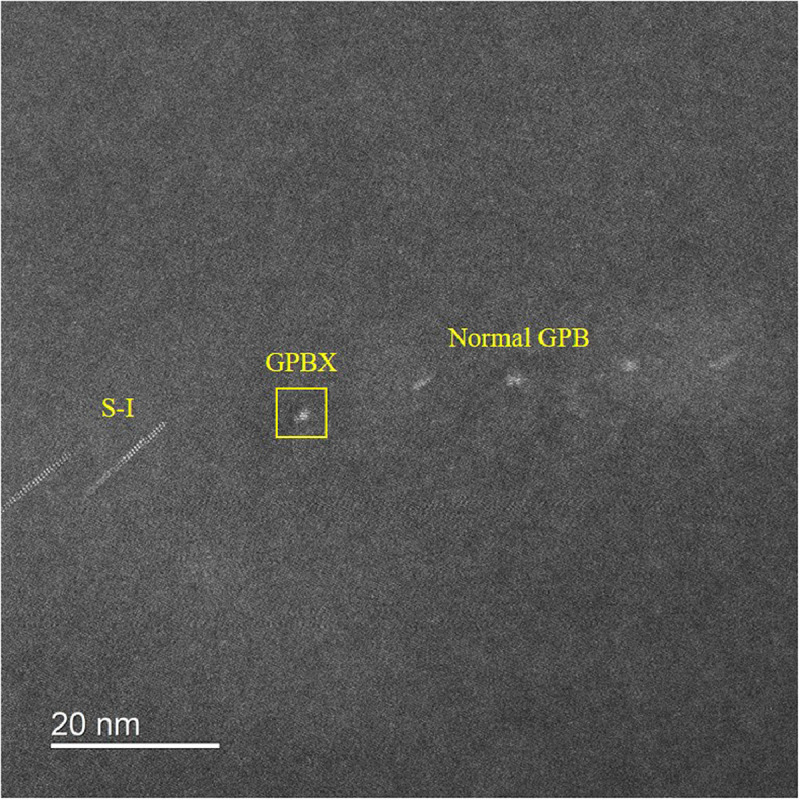
HAADF-STEM image in a <100>_Al_ orientation showing normal GPB zones, novel GPBX zone, and S-I phase along dislocation line in the A.R. samples aged at 443 K for 20 min.

**Fig. 4:**
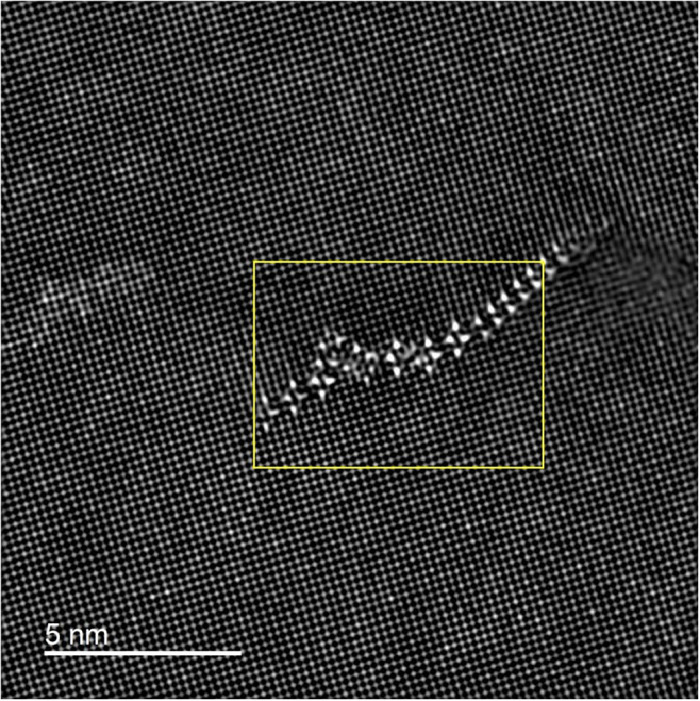
FFT filtered HAADF-STEM image in a <100>_Al_ orientation showing precipitates along dislocation line in the A.R. samples aged at 443 K for 20 min.

Fig. 1 shows the HAADF-STEM images of the as-quenched (A.Q.) and as-rolled (A.R.) samples before aging. There is no precipitate found in the Al matrix (Fig. 1 (a) and (d)) or near the defects (the dislocation loop in Fig. 1(b) and the grain boundary in Fig. 1(c)). Fig. 2 shows two types of S phases [1] formed along dislocations in the A.Q. sample aged at 443 K for 8 h. Fig. 3 and 4 are HAADF-STEM images observed in <100>Al orientation in the 7% cold-rolled Al-3Mg-1Cu alloy aged at 443 K for 20 min. Fig. 3 shows several GPB zones and the GPBX zone as well as the thinnest S-I phase on a dislocation line. The marked area containing the GPBX zone corresponds to Fig. 2(c) of reference [1]. Several GPBX zones and the thinnest S-I phase as well as a forming S-II phase are found to form along a dislocation line as shown in Fig. 4. The selected area is magnified and atomic overlaid to be Fig. 7 of reference [1].

### 1.2 First-principles calculations and atomic models

Table 1 shows the numerical values from DFT calculations of the precipitate structure models presented in Fig. 4 of reference [1], including formation enthalpy per solute atom, pressure and composition. The refined fractional coordinates in the DFT calculated atomic model containing various GPB zones and two types of S phases are listed in Table 2, Table 3, Table 4, Table 5, Table 6, Table 7, Table 8, Table 9, Table 10, Table 11, Table 12, Table 13. In all of the atomic models, the precipitates are embedded in the Al matrix, so the P1 space group is used to give refined coordinates.

**Table 1:** Results from DFT calculations of the precipitate structure models.

	Formation enthalpy per solute atom		Composition		
Refined model	(eV/solute atom)	Pressure (kBar)	Al	Cu	Mg
GPB^1^ (Cu interstitial)	-0.1706	-0.630	135	5	4
GPB^1^ (Al interstitial)	-0.2425	0.720	136	4	4
GPB^2^	-0.2515	1.260	132	6	6
2GPB^2^	-0.2644	2.130	120	12	12
GPBX	-0.1458	14.210	138	4	4
2GPBX_113	-0.1522	26.780	132	8	8
2GPBX_114	-0.1599	27.740	132	8	8
GPBX_relax	-0.2278	0.000	138	4	4
2GPBX_113_relax	-0.2397	-0.020	132	8	8
2GPBX_114_relax	-0.2522	0.000	132	8	8
S-I	-0.2339	6.210	34	4	4
S-II	-0.2171	-33.060	50	12	12

**Table 2:** The refined fractional coordinates in the atomic model for the GPB^1^ (Cu interstitial) zone.

GPB^1^ (Cu interstitial)				a = 24.24 Å, b = 24.24 Å, c = 4.04 Å				α = 90.0°, β = 90.0°, γ = 90.0°			
Atoms	x	y	z	Atoms	x	y	z	Atoms	x	y	z
Al1	0.04162	0.04167	0.25	Al49	0.37466	0.04167	0.25	Al88	0.70842	0.04167	0.25
Al2	0.0418	0.12513	0.75	Al50	0.37473	0.12495	0.75	Al89	0.70826	0.1253	0.75
Al3	0.12489	0.04167	0.75	Al51	0.45833	0.04167	0.75	Al90	0.79178	0.04167	0.75
Al4	0.12511	0.12515	0.25	Al52	0.45833	0.12457	0.25	Al91	0.79156	0.12515	0.25
Al5	0.04181	0.20844	0.25	Al53	0.37459	0.20815	0.25	Al92	0.70822	0.20887	0.25
Al6	0.04196	0.29174	0.75	Al54	0.37539	0.29246	0.75	Al93	0.70759	0.29241	0.75
Al7	0.1252	0.20853	0.75	Al55	0.45833	0.20775	0.75	Al94	0.79147	0.20853	0.75
Al8	0.12554	0.29178	0.25	Al56	0.45833	0.29007	0.25	Al95	0.79113	0.29178	0.25
Al9	0.04181	0.37472	0.25	Al57	0.37748	0.37682	0.25	Al96	0.70717	0.37584	0.25
Al10	0.04162	0.45806	0.75	Mg1	0.38252	0.46585	0.75	Al97	0.70754	0.45872	0.75
Al11	0.1255	0.37488	0.75	Al58	0.45833	0.37511	0.75	Al98	0.79117	0.37488	0.75
Al12	0.12482	0.45792	0.25	Cu1	0.45833	0.45441	0.25	Al99	0.79185	0.45792	0.25
Al13	0.04124	0.54167	0.25	Cu2	0.37108	0.54167	0.25	Al100	0.70993	0.54167	0.25
Al14	0.04162	0.62527	0.75	Mg2	0.38252	0.61748	0.75	Al101	0.70754	0.62461	0.75
Al15	0.12442	0.54167	0.75	Cu3	0.45833	0.54167	0.75	Al102	0.79225	0.54167	0.75
Al16	0.12482	0.62541	0.25	Cu4	0.45833	0.62892	0.25	Al103	0.79185	0.62541	0.25
Al17	0.04181	0.70861	0.25	Al59	0.37748	0.70651	0.25	Al104	0.70717	0.7075	0.25
Al18	0.04196	0.7916	0.75	Al60	0.37539	0.79087	0.75	Al105	0.70759	0.79093	0.75
Al19	0.1255	0.70845	0.75	Al61	0.45833	0.70822	0.75	Al106	0.79117	0.70845	0.75
Al20	0.12554	0.79155	0.25	Al62	0.45833	0.79327	0.25	Al107	0.79113	0.79155	0.25
Al21	0.04181	0.87489	0.25	Al63	0.37459	0.87518	0.25	Al108	0.70822	0.87446	0.25
Al22	0.0418	0.9582	0.75	Al64	0.37473	0.95838	0.75	Al109	0.70826	0.95804	0.75
Al23	0.1252	0.8748	0.75	Al65	0.45833	0.87558	0.75	Al110	0.79147	0.8748	0.75
Al24	0.12511	0.95819	0.25	Al66	0.45833	0.95876	0.25	Al111	0.79156	0.95819	0.25
Al25	0.20825	0.04167	0.25	Al67	0.54201	0.04167	0.25	Al112	0.87505	0.04167	0.25
Al26	0.2084	0.1253	0.75	Al68	0.54194	0.12495	0.75	Al113	0.87487	0.12513	0.75
Al27	0.2914	0.04167	0.75	Al69	0.62527	0.04167	0.75	Al114	0.95833	0.04167	0.75
Al28	0.29139	0.12514	0.25	Al70	0.62528	0.12514	0.25	Al115	0.95833	0.12495	0.25
Al29	0.20845	0.20887	0.25	Al71	0.54208	0.20815	0.25	Al116	0.87485	0.20844	0.25
Al30	0.20907	0.29241	0.75	Al72	0.54128	0.29246	0.75	Al117	0.8747	0.29174	0.75
Al31	0.29155	0.20883	0.75	Al73	0.62512	0.20883	0.75	Al118	0.95833	0.20822	0.75
Al32	0.2925	0.29283	0.25	Al74	0.62417	0.29283	0.25	Al119	0.95833	0.29158	0.25
Al33	0.20949	0.37584	0.25	Al75	0.53919	0.37682	0.25	Al120	0.87486	0.37472	0.25
Al34	0.20913	0.45872	0.75	Mg3	0.53415	0.46585	0.75	Al121	0.87505	0.45806	0.75
Al35	0.2941	0.37744	0.75	Al76	0.62256	0.37744	0.75	Al122	0.95833	0.37473	0.75
Al36	0.29349	0.46081	0.25	Al77	0.62318	0.46081	0.25	Al123	0.95833	0.45799	0.25
Al37	0.20673	0.54167	0.25	Cu5	0.54559	0.54167	0.25	Al124	0.87543	0.54167	0.25
Al38	0.20913	0.62461	0.75	Mg4	0.53415	0.61748	0.75	Al125	0.87505	0.62527	0.75
Al39	0.29178	0.54167	0.75	Al78	0.62489	0.54167	0.75	Al126	0.95833	0.54167	0.75
Al40	0.29349	0.62252	0.25	Al79	0.62318	0.62252	0.25	Al127	0.95833	0.62534	0.25
Al41	0.20949	0.7075	0.25	Al80	0.53919	0.70651	0.25	Al128	0.87486	0.70861	0.25
Al42	0.20907	0.79093	0.75	Al81	0.54128	0.79087	0.75	Al129	0.8747	0.7916	0.75
Al43	0.2941	0.7059	0.75	Al82	0.62256	0.7059	0.75	Al130	0.95833	0.7086	0.75
Al44	0.2925	0.79051	0.25	Al83	0.62417	0.79051	0.25	Al131	0.95833	0.79175	0.25
Al45	0.20845	0.87446	0.25	Al84	0.54208	0.87518	0.25	Al132	0.87485	0.87489	0.25
Al46	0.2084	0.95804	0.75	Al85	0.54194	0.95838	0.75	Al133	0.87487	0.9582	0.75
Al47	0.29155	0.8745	0.75	Al86	0.62512	0.8745	0.75	Al134	0.95833	0.87511	0.75
Al48	0.29139	0.95819	0.25	Al87	0.62528	0.95819	0.25	Al135	0.95833	0.95838	0.25

**Table 3:** The refined fractional coordinates in the atomic model for the GPB^1^ (Al interstitial) zone.

GPB^1^ (Al interstitial)				a = 24.24 Å, b = 24.24 Å, c = 4.04 Å				α = 90.0°, β = 90.0°, γ = 90.0°			
Atoms	x	y	z	Atoms	x	y	z	Atoms	x	y	z
Al1	0.0416	0.04167	0.25	Al49	0.37464	0.04167	0.25	Al89	0.70858	0.04167	0.25
Al2	0.04169	0.12503	0.75	Al50	0.37464	0.1248	0.75	Al90	0.70848	0.12511	0.75
Al3	0.12476	0.04167	0.75	Al51	0.45833	0.04167	0.75	Al91	0.79191	0.04167	0.75
Al4	0.12492	0.12502	0.25	Al52	0.45833	0.12451	0.25	Al92	0.79175	0.12502	0.25
Al5	0.04168	0.20825	0.25	Al53	0.37451	0.20777	0.25	Al93	0.70849	0.20848	0.25
Al6	0.04178	0.29152	0.75	Al54	0.37512	0.29177	0.75	Al94	0.70819	0.29181	0.75
Al7	0.12494	0.20827	0.75	Al55	0.45833	0.20744	0.75	Al95	0.79173	0.20827	0.75
Al8	0.12515	0.29151	0.25	Al56	0.45833	0.28963	0.25	Al96	0.79152	0.29151	0.25
Al9	0.04158	0.37457	0.25	Al57	0.37696	0.37538	0.25	Al97	0.70803	0.37534	0.25
Al10	0.04147	0.45797	0.75	Mg1	0.37883	0.46216	0.75	Al98	0.70823	0.45845	0.75
Al11	0.12499	0.37472	0.75	Al58	0.45833	0.37465	0.75	Al99	0.79168	0.37472	0.75
Al12	0.12443	0.45784	0.25	Cu1	0.45833	0.45518	0.25	Al100	0.79224	0.45784	0.25
Al13	0.04117	0.54167	0.25	Cu2	0.37185	0.54167	0.25	Al101	0.71037	0.54167	0.25
Al14	0.04147	0.62536	0.75	Mg2	0.37883	0.62117	0.75	Al102	0.70823	0.62488	0.75
Al15	0.12411	0.54167	0.75	Al59	0.45833	0.54167	0.75	Al103	0.79256	0.54167	0.75
Al16	0.12443	0.62549	0.25	Cu3	0.45833	0.62815	0.25	Al104	0.79224	0.62549	0.25
Al17	0.04158	0.70877	0.25	Al60	0.37696	0.70795	0.25	Al105	0.70803	0.708	0.25
Al18	0.04178	0.79181	0.75	Al61	0.37512	0.79156	0.75	Al106	0.70819	0.79152	0.75
Al19	0.12499	0.70862	0.75	Al62	0.45833	0.70869	0.75	Al107	0.79168	0.70862	0.75
Al20	0.12515	0.79182	0.25	Al63	0.45833	0.7937	0.25	Al108	0.79152	0.79182	0.25
Al21	0.04168	0.87508	0.25	Al64	0.37451	0.87557	0.25	Al109	0.70849	0.87485	0.25
Al22	0.04169	0.95831	0.75	Al65	0.37464	0.95853	0.75	Al110	0.70848	0.95822	0.75
Al23	0.12494	0.87506	0.75	Al66	0.45833	0.87589	0.75	Al111	0.79173	0.87506	0.75
Al24	0.12492	0.95832	0.25	Al67	0.45833	0.95883	0.25	Al112	0.79175	0.95832	0.25
Al25	0.20808	0.04167	0.25	Al68	0.54202	0.04167	0.25	Al113	0.87507	0.04167	0.25
Al26	0.20819	0.12511	0.75	Al69	0.54203	0.1248	0.75	Al114	0.87497	0.12503	0.75
Al27	0.29123	0.04167	0.75	Al70	0.62544	0.04167	0.75	Al115	0.95833	0.04167	0.75
Al28	0.29123	0.12492	0.25	Al71	0.62543	0.12492	0.25	Al116	0.95833	0.12493	0.25
Al29	0.20818	0.20848	0.25	Al72	0.54216	0.20777	0.25	Al117	0.87498	0.20825	0.25
Al30	0.20848	0.29181	0.75	Al73	0.54155	0.29177	0.75	Al118	0.87489	0.29152	0.75
Al31	0.29138	0.20832	0.75	Al74	0.62528	0.20832	0.75	Al119	0.95833	0.20809	0.75
Al32	0.292	0.29197	0.25	Al75	0.62466	0.29197	0.25	Al120	0.95833	0.29142	0.25
Al33	0.20864	0.37534	0.25	Al76	0.5397	0.37538	0.25	Al121	0.87508	0.37457	0.25
Al34	0.20844	0.45845	0.75	Mg3	0.53784	0.46216	0.75	Al122	0.8752	0.45797	0.75
Al35	0.29269	0.37602	0.75	Al77	0.62398	0.37602	0.75	Al123	0.95833	0.37456	0.75
Al36	0.29205	0.4603	0.25	Al78	0.62462	0.4603	0.25	Al124	0.95833	0.45798	0.25
Al37	0.2063	0.54167	0.25	Cu4	0.54482	0.54167	0.25	Al125	0.87549	0.54167	0.25
Al38	0.20844	0.62488	0.75	Mg4	0.53784	0.62117	0.75	Al126	0.8752	0.62536	0.75
Al39	0.29131	0.54167	0.75	Al79	0.62535	0.54167	0.75	Al127	0.95833	0.54167	0.75
Al40	0.29205	0.62304	0.25	Al80	0.62462	0.62304	0.25	Al128	0.95833	0.62536	0.25
Al41	0.20864	0.708	0.25	Al81	0.5397	0.70795	0.25	Al129	0.87508	0.70877	0.25
Al42	0.20848	0.79152	0.75	Al82	0.54155	0.79156	0.75	Al130	0.87489	0.79181	0.75
Al43	0.29269	0.70731	0.75	Al83	0.62398	0.70731	0.75	Al131	0.95833	0.70877	0.75
Al44	0.292	0.79136	0.25	Al84	0.62466	0.79136	0.25	Al132	0.95833	0.79191	0.25
Al45	0.20818	0.87485	0.25	Al85	0.54216	0.87557	0.25	Al133	0.87498	0.87508	0.25
Al46	0.20819	0.95822	0.75	Al86	0.54203	0.95853	0.75	Al134	0.87497	0.95831	0.75
Al47	0.29138	0.87501	0.75	Al87	0.62528	0.87501	0.75	Al135	0.95833	0.87524	0.75
Al48	0.29123	0.95842	0.25	Al88	0.62543	0.95842	0.25	Al136	0.95833	0.9584	0.25

**Table 4:** The refined fractional coordinates in the atomic model for the GPB^2^ zone.

GPB^2^				a = 24.24 Å, b = 24.24 Å, c = 4.04 Å				α = 90.0°, β = 90.0°, γ = 90.0°			
Atoms	x	y	z	Atoms	x	y	z	Atoms	x	y	z
Al1	0.0418	0.04178	0.25	Al46	0.37488	0.04126	0.25	Al85	0.70859	0.04158	0.25
Al2	0.04189	0.1252	0.75	Al47	0.37479	0.12418	0.75	Al86	0.70857	0.12494	0.75
Al3	0.12493	0.0419	0.75	Al48	0.45837	0.04148	0.75	Al87	0.7918	0.04162	0.75
Al4	0.12521	0.12539	0.25	Al49	0.45841	0.12427	0.25	Al88	0.79173	0.12506	0.25
Al5	0.04217	0.20823	0.25	Al50	0.37478	0.2066	0.25	Al89	0.70822	0.20835	0.25
Al6	0.04206	0.2914	0.75	Al51	0.37506	0.29105	0.75	Al90	0.70786	0.29186	0.75
Al7	0.12555	0.20872	0.75	Al52	0.45788	0.20808	0.75	Al91	0.79158	0.20854	0.75
Al8	0.12586	0.29215	0.25	Al53	0.45662	0.29199	0.25	Al92	0.79167	0.2916	0.25
Al9	0.04152	0.37429	0.25	Cu2	0.37623	0.37381	0.25	Al93	0.70775	0.37536	0.25
Al10	0.04104	0.4582	0.75	Al54	0.37513	0.46118	0.75	Al94	0.70887	0.46003	0.75
Al11	0.12555	0.37496	0.75	Mg3	0.45562	0.37746	0.75	Al95	0.79174	0.37511	0.75
Al12	0.12298	0.45817	0.25	Al55	0.45745	0.46127	0.25	Al96	0.79192	0.45841	0.25
Al13	0.04099	0.5421	0.25	Cu3	0.37708	0.54141	0.25	Al97	0.70884	0.54192	0.25
Al14	0.04149	0.62542	0.75	Al56	0.37564	0.62279	0.75	Al98	0.70722	0.62293	0.75
Al15	0.12475	0.54159	0.75	Al57	0.45922	0.53873	0.75	Al99	0.79368	0.54183	0.75
Al16	0.12492	0.62489	0.25	Mg4	0.46104	0.62254	0.25	Al100	0.79112	0.62504	0.25
Al17	0.04153	0.70872	0.25	Al58	0.37585	0.70922	0.25	Al101	0.70675	0.70694	0.25
Al18	0.04159	0.79179	0.75	Al59	0.37535	0.79225	0.75	Al102	0.70776	0.79104	0.75
Al19	0.125	0.7084	0.75	Al60	0.46004	0.70801	0.75	Al103	0.79081	0.70785	0.75
Al20	0.12508	0.79146	0.25	Al61	0.45879	0.79192	0.25	Al104	0.79111	0.79128	0.25
Al21	0.04153	0.87511	0.25	Al62	0.37489	0.87555	0.25	Al105	0.70845	0.87472	0.25
Al22	0.04144	0.95856	0.75	Al63	0.37486	0.95853	0.75	Al106	0.7087	0.95822	0.75
Al23	0.12493	0.87494	0.75	Al64	0.45825	0.87573	0.75	Al107	0.79146	0.87461	0.75
Al24	0.12486	0.95838	0.25	Al65	0.4583	0.95852	0.25	Al108	0.79174	0.9581	0.25
Al25	0.20797	0.04178	0.25	Al66	0.54181	0.04147	0.25	Al109	0.87523	0.04144	0.25
Al26	0.20822	0.12528	0.75	Al67	0.54178	0.12445	0.75	Al110	0.87513	0.12489	0.75
Al27	0.29137	0.04161	0.75	Al68	0.62523	0.04158	0.75	Al111	0.95854	0.04163	0.75
Al28	0.29131	0.12474	0.25	Al69	0.62517	0.12476	0.25	Al112	0.95854	0.12505	0.25
Al29	0.20891	0.20895	0.25	Al70	0.54132	0.20775	0.25	Al113	0.87507	0.20821	0.25
Al30	0.20992	0.29306	0.75	Al71	0.54081	0.29078	0.75	Al114	0.87514	0.29128	0.75
Al31	0.29179	0.20857	0.75	Al72	0.625	0.20789	0.75	Al115	0.95865	0.20825	0.75
Al32	0.29398	0.29252	0.25	Al73	0.62452	0.29199	0.25	Al116	0.95849	0.29131	0.25
Al33	0.20944	0.37706	0.25	Al74	0.54103	0.37721	0.25	Al117	0.87518	0.37458	0.25
Al34	0.20783	0.45808	0.75	Cu4	0.53958	0.45859	0.75	Al118	0.87567	0.4579	0.75
Mg1	0.29657	0.37948	0.75	Al75	0.62205	0.37551	0.75	Al119	0.95857	0.37447	0.75
Cu1	0.28912	0.45909	0.25	Mg5	0.62293	0.46158	0.25	Al120	0.95812	0.4581	0.25
Al35	0.20779	0.53997	0.25	Al76	0.54153	0.53882	0.25	Al121	0.87563	0.5418	0.25
Al36	0.20892	0.62464	0.75	Cu5	0.54044	0.62619	0.75	Al122	0.87514	0.62571	0.75
Mg2	0.29374	0.53842	0.75	Cu6	0.62754	0.54091	0.75	Al123	0.95855	0.5419	0.75
Al37	0.29462	0.62449	0.25	Mg6	0.6201	0.62052	0.25	Al124	0.9581	0.62553	0.25
Al38	0.2088	0.70814	0.25	Al77	0.54161	0.70895	0.25	Al125	0.87461	0.7086	0.25
Al39	0.20844	0.79165	0.75	Al78	0.54188	0.7934	0.75	Al126	0.8745	0.79177	0.75
Al40	0.29215	0.70801	0.75	Al79	0.62268	0.70748	0.75	Al127	0.95817	0.70869	0.75
Al41	0.29167	0.79211	0.25	Al80	0.62488	0.79143	0.25	Al128	0.95802	0.79175	0.25
Al42	0.2081	0.87506	0.25	Al81	0.54188	0.87583	0.25	Al129	0.87478	0.8748	0.25
Al43	0.20808	0.95842	0.75	Al82	0.54179	0.95874	0.75	Al130	0.87486	0.95822	0.75
Al44	0.29149	0.87524	0.75	Al83	0.62535	0.87526	0.75	Al131	0.95812	0.87495	0.75
Al45	0.29144	0.95842	0.25	Al84	0.62529	0.95839	0.25	Al132	0.95813	0.95837	0.25

**Table 5:** The refined fractional coordinates in the atomic model for the 2GPB^2^ zone.

2GPB^2^				a = 24.24 Å, b = 24.24 Å, c = 4.04 Å				α = 90.0°, β = 90.0°, γ = 90.0°			
Atoms	x	y	z	Atoms	x	y	z	Atoms	x	y	z
Al1	0.04174	0.04169	0.25	Al43	0.37505	0.04235	0.25	Al79	0.70879	0.04101	0.25
Al2	0.04161	0.12507	0.75	Al44	0.37588	0.12527	0.75	Al80	0.70817	0.1248	0.75
Al3	0.12474	0.04154	0.75	Al45	0.45853	0.04187	0.75	Al81	0.79221	0.04151	0.75
Al4	0.12477	0.12506	0.25	Al46	0.45932	0.12593	0.25	Al82	0.79151	0.12493	0.25
Al5	0.04194	0.2085	0.25	Al47	0.37571	0.20911	0.25	Al83	0.70655	0.208	0.25
Al6	0.04202	0.29167	0.75	Al48	0.37375	0.29218	0.75	Mg10	0.70487	0.29373	0.75
Al7	0.12552	0.20878	0.75	Al49	0.45976	0.21042	0.75	Al84	0.79101	0.20886	0.75
Al8	0.12618	0.29271	0.25	Al50	0.45855	0.29495	0.25	Al85	0.79091	0.29418	0.25
Al9	0.04209	0.37467	0.25	Mg4	0.37047	0.3777	0.25	Cu10	0.70846	0.37695	0.25
Al10	0.04021	0.45803	0.75	Cu4	0.37505	0.45981	0.75	Al86	0.7057	0.45973	0.75
Al11	0.12546	0.37695	0.75	Al51	0.45691	0.37776	0.75	Al87	0.78916	0.37524	0.75
Al12	0.12438	0.45858	0.25	Al52	0.45682	0.45755	0.25	Mg11	0.78944	0.46056	0.25
Al13	0.04238	0.54163	0.25	Al53	0.37169	0.54144	0.25	Al88	0.70669	0.54172	0.25
Al14	0.04193	0.62537	0.75	Al54	0.37174	0.62434	0.75	Cu11	0.70879	0.62693	0.75
Al15	0.12551	0.54052	0.75	Mg5	0.45542	0.54388	0.75	Cu12	0.79384	0.54033	0.75
Al16	0.12459	0.62495	0.25	Cu5	0.45968	0.62319	0.25	Mg12	0.78788	0.62049	0.25
Al17	0.04152	0.70881	0.25	Cu6	0.37443	0.71072	0.25	Al89	0.7086	0.70891	0.25
Al18	0.04172	0.79158	0.75	Al55	0.37545	0.79176	0.75	Al90	0.708	0.7933	0.75
Al19	0.12584	0.70823	0.75	Mg6	0.45431	0.70231	0.75	Al91	0.78998	0.70738	0.75
Al20	0.12548	0.79121	0.25	Al56	0.45628	0.79011	0.25	Al92	0.79149	0.79129	0.25
Al21	0.04178	0.8749	0.25	Al57	0.37496	0.87692	0.25	Al93	0.70815	0.87521	0.25
Al22	0.04149	0.95821	0.75	Al58	0.37499	0.95905	0.75	Al94	0.70844	0.95834	0.75
Al23	0.12508	0.8749	0.75	Al59	0.45796	0.87437	0.75	Al95	0.79208	0.87531	0.75
Al24	0.12462	0.95811	0.25	Al60	0.45845	0.95869	0.25	Al96	0.79209	0.95815	0.25
Al25	0.20791	0.04185	0.25	Al61	0.54155	0.04131	0.25	Al97	0.87538	0.04189	0.25
Al26	0.20792	0.12469	0.75	Al62	0.54204	0.12563	0.75	Al98	0.87492	0.1251	0.75
Al27	0.29156	0.04166	0.75	Al63	0.62501	0.04095	0.75	Al99	0.95851	0.04179	0.75
Al28	0.29185	0.12479	0.25	Al64	0.62504	0.12308	0.25	Al100	0.95822	0.1251	0.25
Al29	0.20851	0.20871	0.25	Al65	0.54372	0.20989	0.25	Al101	0.87452	0.20879	0.25
Al30	0.21002	0.29262	0.75	Mg7	0.54569	0.29769	0.75	Al102	0.87416	0.29177	0.75
Al31	0.292	0.2067	0.75	Al66	0.62455	0.20824	0.75	Al103	0.95828	0.20842	0.75
Al32	0.2914	0.29109	0.25	Cu7	0.62557	0.28929	0.25	Al104	0.95848	0.29119	0.25
Mg1	0.21212	0.37951	0.25	Cu8	0.54032	0.37681	0.25	Al105	0.87541	0.37505	0.25
Cu1	0.20616	0.45967	0.75	Mg8	0.54458	0.45612	0.75	Al106	0.87449	0.45948	0.75
Cu2	0.29121	0.37307	0.75	Al67	0.62826	0.37566	0.75	Al107	0.95807	0.37463	0.75
Al33	0.29331	0.45828	0.25	Al68	0.62831	0.45856	0.25	Al108	0.95762	0.45837	0.25
Mg2	0.21056	0.53944	0.25	Al69	0.54318	0.54245	0.25	Al109	0.87562	0.54142	0.25
Al34	0.21084	0.62476	0.75	Al70	0.54309	0.62224	0.75	Al110	0.87454	0.62305	0.75
Al35	0.2943	0.54027	0.75	Cu9	0.62495	0.54019	0.75	Al111	0.95979	0.54197	0.75
Cu3	0.29154	0.62305	0.25	Mg9	0.62953	0.6223	0.25	Al112	0.95791	0.62533	0.25
Al36	0.20909	0.70582	0.25	Al71	0.54145	0.70505	0.25	Al113	0.87382	0.70729	0.25
Al37	0.20899	0.79114	0.75	Al72	0.54024	0.78958	0.75	Al114	0.87448	0.79122	0.75
Mg3	0.29513	0.70627	0.75	Al73	0.62625	0.70782	0.75	Al115	0.95798	0.70833	0.75
Al38	0.29345	0.792	0.25	Al74	0.62429	0.79089	0.25	Al116	0.95806	0.7915	0.25
Al39	0.20849	0.87507	0.25	Al75	0.54068	0.87407	0.25	Al117	0.87523	0.87493	0.25
Al40	0.20779	0.95849	0.75	Al76	0.54147	0.95813	0.75	Al118	0.87526	0.95846	0.75
Al41	0.29183	0.8752	0.75	Al77	0.62412	0.87472	0.75	Al119	0.95839	0.87493	0.75
Al42	0.29121	0.95899	0.25	Al78	0.62495	0.95765	0.25	Al120	0.95826	0.95831	0.25

**Table 6:** The refined fractional coordinates in the atomic model for the GPBX zone.

GPBX				a = 24.24 Å, b = 24.24 Å, c = 4.04 Å				α = 90.0°, β = 90.0°, γ = 90.0°			
Atoms	x	y	z	Atoms	x	y	z	Atoms	x	y	z
Al1	0.03892	0.04266	0.25	Al50	0.70722	0.79094	0.75	Al98	0.29334	0.35722	0.75
Al2	0.03955	0.12523	0.75	Al51	0.79005	0.70834	0.75	Cu1	0.29549	0.41554	0.25
Al3	0.122	0.04279	0.75	Al52	0.78993	0.79154	0.25	Al99	0.19896	0.5525	0.25
Al4	0.12252	0.12445	0.25	Al53	0.7084	0.87435	0.25	Al100	0.2	0.63915	0.75
Al5	0.04044	0.20831	0.25	Al54	0.70923	0.9577	0.75	Cu2	0.26187	0.56079	0.75
Al6	0.0422	0.29221	0.75	Al55	0.79063	0.87473	0.75	Mg2	0.29685	0.63344	0.25
Al7	0.12319	0.207	0.75	Al56	0.79135	0.95784	0.25	Al101	0.20428	0.71779	0.25
Al8	0.12391	0.28988	0.25	Al57	0.87448	0.04201	0.25	Al102	0.20355	0.80065	0.75
Al9	0.20598	0.0423	0.25	Al58	0.87531	0.12545	0.75	Al103	0.2851	0.72282	0.75
Al10	0.20638	0.12276	0.75	Al59	0.95663	0.0424	0.75	Al104	0.28864	0.80271	0.25
Al11	0.29078	0.04171	0.75	Al60	0.95742	0.12559	0.25	Al105	0.20466	0.881	0.25
Al12	0.29112	0.12175	0.25	Al61	0.87602	0.20858	0.25	Al106	0.20514	0.96177	0.75
Al13	0.20681	0.20376	0.25	Al62	0.87671	0.29178	0.75	Al107	0.28955	0.88221	0.75
Al14	0.20712	0.28496	0.75	Al63	0.95824	0.20896	0.75	Al108	0.29009	0.96212	0.25
Al15	0.29177	0.20101	0.75	Al64	0.95943	0.29238	0.25	Al109	0.38156	0.36053	0.25
Al16	0.29242	0.28032	0.25	Al65	0.87663	0.37497	0.25	Mg3	0.36982	0.44988	0.75
Al17	0.37587	0.04164	0.25	Al66	0.87616	0.45836	0.75	Al110	0.46236	0.36555	0.75
Al18	0.37655	0.12123	0.75	Al67	0.95945	0.37564	0.75	Al111	0.46668	0.44418	0.25
Al19	0.46069	0.04105	0.75	Al68	0.95881	0.45891	0.25	Cu3	0.40483	0.52252	0.25
Al20	0.46151	0.12158	0.25	Al69	0.87445	0.54193	0.25	Cu4	0.37119	0.6678	0.75
Al21	0.37712	0.20113	0.25	Al70	0.87334	0.62564	0.75	Al112	0.46772	0.53084	0.75
Al22	0.378	0.28063	0.75	Al71	0.95737	0.54241	0.75	Mg4	0.44281	0.62517	0.25
Al23	0.46199	0.20236	0.75	Al72	0.95552	0.62644	0.25	Al113	0.37331	0.72612	0.25
Al24	0.4631	0.28269	0.25	Al73	0.87271	0.70915	0.25	Al114	0.37424	0.80302	0.75
Al25	0.54466	0.04056	0.25	Al74	0.8724	0.79236	0.75	Al115	0.46121	0.7149	0.75
Al26	0.54551	0.12216	0.75	Al75	0.95489	0.71033	0.75	Al116	0.45953	0.7984	0.25
Al27	0.62774	0.04067	0.75	Al76	0.95471	0.79382	0.25	Al117	0.37486	0.88234	0.25
Al28	0.62855	0.12282	0.25	Al77	0.87283	0.87556	0.25	Al118	0.37553	0.9616	0.75
Al29	0.54635	0.20383	0.25	Al78	0.87362	0.95873	0.75	Al119	0.45984	0.8796	0.75
Al30	0.54612	0.28612	0.75	Al79	0.95501	0.87682	0.75	Al120	0.46027	0.96059	0.25
Al31	0.62919	0.20528	0.75	Al80	0.95581	0.95956	0.25	Al121	0.54792	0.36816	0.25
Al32	0.62971	0.28762	0.25	Al81	0.04259	0.3759	0.25	Al122	0.54815	0.45215	0.75
Al33	0.71005	0.0409	0.25	Al82	0.04251	0.45932	0.75	Al123	0.62964	0.37123	0.75
Al34	0.71086	0.12374	0.75	Al83	0.12602	0.37637	0.75	Al124	0.62927	0.45542	0.25
Al35	0.79221	0.04127	0.75	Al84	0.12686	0.46018	0.25	Al125	0.5457	0.53873	0.25
Al36	0.79307	0.12457	0.25	Al85	0.03911	0.54339	0.25	Al126	0.5398	0.62317	0.75
Al37	0.71166	0.2065	0.25	Al86	0.03741	0.62792	0.75	Al127	0.62759	0.53995	0.75
Al38	0.71194	0.28951	0.75	Al87	0.121	0.54461	0.75	Al128	0.62419	0.624	0.25
Al39	0.79382	0.20776	0.75	Al88	0.11854	0.63119	0.25	Al129	0.54064	0.70695	0.25
Al40	0.79425	0.29097	0.25	Al89	0.03705	0.71212	0.25	Al130	0.54274	0.79346	0.75
Al41	0.7118	0.37299	0.25	Al90	0.03695	0.79573	0.75	Al131	0.62407	0.70742	0.75
Al42	0.71117	0.4569	0.75	Al91	0.11874	0.71519	0.75	Al132	0.62445	0.7911	0.25
Al43	0.79397	0.37419	0.75	Al92	0.12052	0.79725	0.25	Al133	0.54345	0.87635	0.25
Al44	0.79336	0.45769	0.25	Al93	0.03746	0.87806	0.25	Al134	0.54413	0.9589	0.75
Al45	0.70934	0.54091	0.25	Al94	0.03812	0.9605	0.75	Al135	0.62621	0.875	0.75
Al46	0.70789	0.62441	0.75	Al95	0.12032	0.87953	0.75	Al136	0.6271	0.95809	0.25
Al47	0.79228	0.54139	0.75	Al96	0.12113	0.96121	0.25	Al137	0.30332	0.51411	0.25
Al48	0.79054	0.62496	0.25	Al97	0.20546	0.36845	0.25	Al138	0.36336	0.5692	0.75
Al49	0.70722	0.70767	0.25	Mg1	0.22384	0.45818	0.75

**Table 7:** The refined fractional coordinates in the atomic model for the 2GPBX_113 zone.

2GPBX_113				a = 24.24 Å, b = 24.24 Å, c = 4.04 Å				α = 90.0°, β = 90.0°, γ = 90.0°			
Atoms	x	y	z	Atoms	x	y	z	Atoms	x	y	z
Al1	0.03899	0.04219	0.25	Al51	0.37142	0.96552	0.75	Al101	0.872	0.70766	0.25
Al2	0.04037	0.12502	0.75	Al52	0.45565	0.88755	0.75	Al102	0.87155	0.79083	0.75
Al3	0.12088	0.04298	0.75	Al53	0.4571	0.96594	0.25	Al103	0.95486	0.70924	0.75
Al4	0.1222	0.12489	0.25	Al54	0.54324	0.04416	0.25	Al104	0.95391	0.79256	0.25
Al5	0.04213	0.20826	0.25	Al55	0.54468	0.12255	0.75	Al105	0.87256	0.87468	0.25
Al6	0.04452	0.29201	0.75	Al56	0.62836	0.0437	0.75	Al106	0.87496	0.95847	0.75
Al7	0.12374	0.20782	0.75	Al57	0.62946	0.12307	0.25	Al107	0.95438	0.8758	0.75
Al8	0.12533	0.29051	0.25	Al58	0.5457	0.20118	0.25	Al108	0.95611	0.95863	0.25
Al9	0.04557	0.37583	0.25	Al59	0.54575	0.27981	0.75	Mg1	0.22584	0.45986	0.75
Al10	0.04511	0.45898	0.75	Al60	0.63057	0.20219	0.75	Al109	0.29422	0.35849	0.75
Al11	0.12828	0.37709	0.75	Al61	0.63145	0.28153	0.25	Cu1	0.29694	0.41698	0.25
Al12	0.12937	0.46062	0.25	Al62	0.54063	0.88685	0.25	Cu2	0.26484	0.56249	0.75
Al13	0.04149	0.54293	0.25	Al63	0.54226	0.96566	0.75	Mg2	0.29688	0.63442	0.25
Al14	0.03863	0.62746	0.75	Al64	0.62581	0.88555	0.75	Al110	0.28275	0.72483	0.75
Al15	0.123	0.54458	0.75	Al65	0.62738	0.96487	0.25	Al111	0.28554	0.80528	0.25
Al16	0.11938	0.63094	0.25	Al66	0.71225	0.04288	0.25	Al112	0.38097	0.35923	0.25
Al17	0.03672	0.71145	0.25	Al67	0.7133	0.12341	0.75	Mg3	0.37232	0.45029	0.75
Al18	0.03601	0.79496	0.75	Al68	0.79486	0.04183	0.75	Al113	0.46168	0.36035	0.75
Al19	0.11819	0.71493	0.75	Al69	0.7962	0.12374	0.25	Al114	0.46515	0.43803	0.25
Al20	0.11908	0.79697	0.25	Al70	0.71481	0.20377	0.25	Cu3	0.40709	0.52075	0.25
Al21	0.03643	0.87729	0.25	Al71	0.71537	0.28446	0.75	Cu4	0.36794	0.66889	0.75
Al22	0.03745	0.95977	0.75	Al72	0.79755	0.20552	0.75	Mg4	0.43492	0.63083	0.25
Al23	0.11854	0.87936	0.75	Al73	0.79854	0.28734	0.25	Al115	0.36847	0.73045	0.25
Al24	0.11953	0.96119	0.25	Al74	0.798	0.36974	0.75	Al116	0.36971	0.80876	0.75
Al25	0.20376	0.04331	0.25	Al75	0.79891	0.45181	0.25	Al117	0.45198	0.72874	0.75
Al26	0.20483	0.12383	0.75	Al76	0.71604	0.61365	0.75	Al118	0.45546	0.80638	0.25
Al27	0.28762	0.04364	0.75	Al77	0.79772	0.53578	0.75	Al119	0.30556	0.51531	0.25
Al28	0.28872	0.123	0.25	Al78	0.79407	0.62209	0.25	Al120	0.36728	0.56866	0.75
Al29	0.20603	0.20488	0.25	Al79	0.78774	0.70607	0.75	Mg5	0.48216	0.53589	0.75
Al30	0.20748	0.2864	0.75	Al80	0.78885	0.78959	0.25	Al121	0.54866	0.43629	0.75
Al31	0.28972	0.20181	0.75	Al81	0.70965	0.88031	0.25	Cu5	0.54922	0.49784	0.25
Al32	0.29131	0.28114	0.25	Al82	0.71107	0.96182	0.75	Cu6	0.50996	0.64599	0.75
Al33	0.20701	0.36981	0.25	Al83	0.79178	0.87619	0.75	Mg6	0.54481	0.71644	0.25
Al34	0.20103	0.55306	0.25	Al84	0.79337	0.9589	0.25	Al122	0.53618	0.80749	0.75
Al35	0.20049	0.6393	0.75	Al85	0.8767	0.04171	0.25	Al123	0.54741	0.35797	0.25
Al36	0.20276	0.71834	0.25	Al86	0.87809	0.12453	0.75	Al124	0.63439	0.44191	0.25
Al37	0.20121	0.80155	0.75	Al87	0.95773	0.04181	0.75	Mg7	0.62022	0.53231	0.75
Al38	0.20173	0.88226	0.25	Al88	0.95935	0.1249	0.25	Al125	0.71437	0.44841	0.75
Al39	0.20227	0.96295	0.75	Al89	0.87963	0.20694	0.25	Al126	0.71661	0.52744	0.25
Al40	0.28566	0.8852	0.75	Al90	0.88065	0.28943	0.75	Cu7	0.65224	0.60424	0.25
Al41	0.28652	0.96454	0.25	Al91	0.96097	0.20809	0.75	Cu8	0.62021	0.74975	0.75
Al42	0.37241	0.04414	0.25	Al92	0.96269	0.2909	0.25	Mg8	0.6913	0.70685	0.25
Al43	0.37387	0.12254	0.75	Al93	0.88106	0.37174	0.25	Al127	0.62293	0.80823	0.25
Al44	0.45778	0.04417	0.75	Al94	0.88039	0.45522	0.75	Al128	0.63157	0.36145	0.75
Al45	0.45933	0.12253	0.25	Al95	0.96318	0.37413	0.75	Al129	0.71014	0.79689	0.75
Al46	0.37486	0.20104	0.25	Al96	0.96227	0.45741	0.25	Al130	0.7159	0.36519	0.25
Al47	0.3765	0.27987	0.75	Al97	0.87847	0.53918	0.25	Al131	0.54978	0.59807	0.25
Al48	0.46001	0.20077	0.75	Al98	0.8756	0.62372	0.75	Al132	0.61151	0.65142	0.75
Al49	0.46149	0.27916	0.25	Al99	0.96014	0.54118	0.75				
Al50	0.37138	0.88692	0.25	Al100	0.95695	0.62546	0.25

**Table 8:** The refined fractional coordinates in the atomic model for the 2GPBX_114 zone.

2GPBX_114				a = 24.24 Å, b = 24.24 Å, c = 4.04 Å				α = 90.0°, β = 90.0°, γ = 90.0°			
Atoms	x	y	z	Atoms	x	y	z	Atoms	x	y	z
Al1	0.03984	0.0424	0.25	Al51	0.62617	0.04438	0.75	Al99	0.20077	0.55277	0.25
Al2	0.0412	0.1247	0.75	Al52	0.62762	0.12331	0.25	Al100	0.19969	0.63907	0.75
Al3	0.12161	0.04256	0.75	Al53	0.54422	0.20228	0.25	Cu2	0.26356	0.56201	0.75
Al4	0.12294	0.12439	0.25	Al54	0.54433	0.28239	0.75	Mg2	0.29586	0.63456	0.25
Al5	0.04287	0.20749	0.25	Al55	0.62842	0.20264	0.75	Al101	0.20234	0.71807	0.25
Al6	0.04513	0.29079	0.75	Al56	0.62942	0.28122	0.25	Al102	0.20121	0.80112	0.75
Al7	0.12455	0.20699	0.75	Al57	0.53865	0.88517	0.25	Al103	0.28231	0.7243	0.75
Al8	0.12593	0.28968	0.25	Al58	0.54058	0.96476	0.75	Al104	0.28548	0.80455	0.25
Al9	0.04609	0.37437	0.25	Al59	0.62326	0.8858	0.75	Al105	0.38177	0.36182	0.25
Al10	0.04593	0.45794	0.75	Al60	0.62504	0.9651	0.25	Mg3	0.3715	0.45147	0.75
Al11	0.12853	0.37633	0.75	Al61	0.7107	0.04409	0.25	Al106	0.46142	0.36457	0.75
Al12	0.12916	0.46016	0.25	Al62	0.71169	0.12396	0.75	Al107	0.46774	0.44518	0.25
Al13	0.04207	0.54242	0.25	Al63	0.79424	0.04356	0.75	Cu3	0.40759	0.52459	0.25
Al14	0.03834	0.62742	0.75	Al64	0.7954	0.12427	0.25	Cu4	0.36736	0.66934	0.75
Al15	0.12309	0.54451	0.75	Al65	0.71298	0.20335	0.25	Al108	0.47018	0.53317	0.75
Al16	0.11918	0.63086	0.25	Al66	0.71383	0.28302	0.75	Mg4	0.43895	0.6287	0.25
Al17	0.03651	0.71148	0.25	Al67	0.79683	0.20486	0.75	Al109	0.36938	0.72872	0.25
Al18	0.03593	0.79503	0.75	Al68	0.79772	0.2857	0.25	Al110	0.37047	0.80645	0.75
Al19	0.11776	0.71494	0.75	Al69	0.70802	0.8853	0.25	Al111	0.45448	0.72181	0.75
Al20	0.11897	0.79705	0.25	Al70	0.70938	0.96476	0.75	Al112	0.45372	0.80482	0.25
Al21	0.03661	0.8775	0.25	Al71	0.79204	0.88112	0.75	Al113	0.3056	0.51549	0.25
Al22	0.03823	0.96011	0.75	Al72	0.79299	0.96231	0.25	Al114	0.365	0.57041	0.75
Al23	0.1188	0.8793	0.75	Al73	0.87689	0.04279	0.25	Al115	0.5454	0.44537	0.75
Al24	0.12016	0.96096	0.25	Al74	0.87822	0.12464	0.75	Mg5	0.56093	0.53848	0.25
Al25	0.20443	0.04291	0.25	Al75	0.95863	0.04249	0.75	Al116	0.6305	0.43845	0.25
Al26	0.2056	0.12361	0.75	Al76	0.95997	0.12481	0.25	Cu5	0.63252	0.49781	0.75
Al27	0.28816	0.04323	0.75	Al77	0.87968	0.20624	0.25	Al117	0.52971	0.63402	0.75
Al28	0.28914	0.12309	0.25	Al78	0.88107	0.28789	0.75	Al118	0.53213	0.72202	0.25
Al29	0.20685	0.20486	0.25	Al79	0.96161	0.20708	0.75	Cu6	0.59228	0.64261	0.25
Al30	0.2078	0.28605	0.75	Al80	0.96325	0.28969	0.25	Mg6	0.62834	0.71572	0.75
Al31	0.29046	0.20242	0.75	Al81	0.88091	0.37016	0.25	Al119	0.53843	0.80264	0.75
Al32	0.2918	0.28189	0.25	Al82	0.88212	0.45227	0.75	Al120	0.54616	0.36238	0.25
Al33	0.20216	0.88152	0.25	Al83	0.96392	0.37216	0.75	Al121	0.61808	0.80539	0.25
Al34	0.20303	0.96235	0.75	Al84	0.96336	0.45572	0.25	Al122	0.62942	0.36073	0.75
Al35	0.28606	0.88418	0.75	Al85	0.8807	0.53635	0.25	Al123	0.71759	0.44289	0.75
Al36	0.28688	0.96385	0.25	Al86	0.87676	0.62269	0.75	Mg7	0.70402	0.53262	0.25
Al37	0.37222	0.0439	0.25	Al87	0.96155	0.53978	0.75	Al124	0.79754	0.44914	0.25
Al38	0.37369	0.12281	0.75	Al88	0.95781	0.62478	0.25	Al125	0.80019	0.52814	0.75
Al39	0.45676	0.04399	0.75	Al89	0.87071	0.70703	0.25	Cu7	0.73631	0.60521	0.75
Al40	0.45838	0.12321	0.25	Al90	0.87131	0.79083	0.75	Cu8	0.70348	0.75015	0.25
Al41	0.3748	0.20211	0.25	Al91	0.95394	0.70923	0.75	Al126	0.7991	0.61445	0.25
Al42	0.37658	0.2814	0.75	Al92	0.95377	0.79282	0.25	Mg8	0.77426	0.70784	0.75
Al43	0.45927	0.20244	0.75	Al93	0.87391	0.87749	0.25	Al127	0.70574	0.80836	0.75
Al44	0.46119	0.28203	0.25	Al94	0.87529	0.96016	0.75	Al128	0.71441	0.36265	0.25
Al45	0.37045	0.88596	0.25	Al95	0.9547	0.87639	0.75	Al129	0.79296	0.79784	0.25
Al46	0.37143	0.96456	0.75	Al96	0.95697	0.9597	0.25	Al130	0.79869	0.36609	0.75
Al47	0.45552	0.88481	0.75	Al97	0.20688	0.36934	0.25	Al131	0.63487	0.59675	0.75
Al48	0.45562	0.96493	0.25	Mg1	0.22556	0.45937	0.75	Al132	0.69426	0.6517	0.25
Al49	0.54146	0.044	0.25	Al98	0.29408	0.35883	0.75				
Al50	0.54309	0.1232	0.75	Cu1	0.29636	0.41704	0.25

**Table 9:** The refined fractional coordinates in the atomic model for the GPBX_relax zone.

GPBX_relax				a = 24.49 Å, b = 24.49 Å, c = 3.97 Å				α = 90.0°, β = 90.0°, γ = 90.6°			
Atoms	x	y	z	Atoms	x	y	z	Atoms	x	y	z
Al1	0.0392	0.04248	0.25	Al50	0.70683	0.79076	0.75	Al99	0.19861	0.55269	0.25
Al2	0.03986	0.12542	0.75	Al51	0.78969	0.70826	0.75	Al100	0.20017	0.63872	0.75
Al3	0.12208	0.04269	0.75	Al52	0.78974	0.79131	0.25	Al101	0.20449	0.71767	0.25
Al4	0.12267	0.12472	0.25	Al53	0.70816	0.8741	0.25	Al102	0.20392	0.79995	0.75
Al5	0.04093	0.20885	0.25	Al54	0.70911	0.95768	0.75	Al103	0.28601	0.72292	0.75
Al6	0.04273	0.29278	0.75	Al55	0.79056	0.87451	0.75	Al104	0.28896	0.80245	0.25
Al7	0.12349	0.20768	0.75	Al56	0.79133	0.95786	0.25	Al105	0.20467	0.88036	0.25
Al8	0.12468	0.29085	0.25	Al57	0.87445	0.04207	0.25	Al106	0.20509	0.9614	0.75
Al9	0.2058	0.04204	0.25	Al58	0.87531	0.12547	0.75	Al107	0.28935	0.88217	0.75
Al10	0.20639	0.12272	0.75	Al59	0.95671	0.04237	0.75	Al108	0.29007	0.96205	0.25
Al11	0.29068	0.04156	0.75	Al60	0.95752	0.12566	0.25	Al109	0.38067	0.36041	0.25
Al12	0.29118	0.12137	0.25	Al61	0.87609	0.20882	0.25	Al110	0.46217	0.36566	0.75
Al13	0.20698	0.20387	0.25	Al62	0.87693	0.29201	0.75	Al111	0.4665	0.44461	0.25
Al14	0.20785	0.2854	0.75	Al63	0.9585	0.20923	0.75	Al112	0.46808	0.53066	0.74999
Al15	0.29196	0.20054	0.75	Al64	0.95985	0.29257	0.25	Al113	0.37295	0.726	0.24999
Al16	0.29281	0.27944	0.25	Al65	0.87698	0.37507	0.25	Al114	0.37387	0.8039	0.75
Al17	0.37598	0.04179	0.25	Al66	0.87637	0.45825	0.75	Al115	0.45989	0.71469	0.74999
Al18	0.37658	0.1213	0.75	Al67	0.96006	0.37573	0.75	Al116	0.45883	0.79792	0.25
Al19	0.46084	0.0413	0.75	Al68	0.95926	0.45891	0.25	Al117	0.3747	0.88281	0.25
Al20	0.46156	0.12192	0.25	Al69	0.87436	0.54204	0.25	Al118	0.37549	0.96198	0.75
Al21	0.37732	0.20117	0.25	Al70	0.87292	0.62574	0.75	Al119	0.45968	0.87946	0.75
Al22	0.37773	0.28088	0.75	Al71	0.95715	0.54261	0.75	Al120	0.46027	0.96061	0.25
Al23	0.46201	0.20296	0.75	Al72	0.95522	0.6265	0.25	Al121	0.54729	0.36872	0.25
Al24	0.46275	0.28338	0.25	Al73	0.87239	0.70911	0.25	Al122	0.54809	0.4527	0.75
Al25	0.54456	0.04064	0.25	Al74	0.87226	0.79217	0.75	Al123	0.62916	0.3715	0.75
Al26	0.54524	0.12256	0.75	Al75	0.95484	0.71023	0.75	Al124	0.62949	0.4553	0.25
Al27	0.62744	0.04085	0.75	Al76	0.95476	0.79335	0.25	Al125	0.54662	0.53828	0.25
Al28	0.62813	0.12319	0.25	Al77	0.87286	0.87529	0.25	Al126	0.53928	0.62272	0.74999
Al29	0.54597	0.2045	0.25	Al78	0.87362	0.95871	0.75	Al127	0.62796	0.53974	0.75
Al30	0.54591	0.2869	0.75	Al79	0.95525	0.87625	0.75	Al128	0.62353	0.62375	0.25
Al31	0.62888	0.20586	0.75	Al80	0.95592	0.95927	0.25	Al129	0.53988	0.70632	0.24999
Al32	0.62924	0.28821	0.25	Al81	0.04338	0.37632	0.25	Al130	0.542	0.79248	0.75
Al33	0.70994	0.04097	0.25	Al82	0.04313	0.45959	0.75	Al131	0.62332	0.70703	0.75
Al34	0.71072	0.12406	0.75	Al83	0.12681	0.37702	0.75	Al132	0.62396	0.79058	0.25
Al35	0.7922	0.04125	0.75	Al84	0.12739	0.46063	0.25	Al133	0.54317	0.87564	0.25
Al36	0.79303	0.12461	0.25	Al85	0.03871	0.54359	0.25	Al134	0.54397	0.95862	0.75
Al37	0.71141	0.20708	0.25	Al86	0.03718	0.62803	0.75	Al135	0.62573	0.8745	0.75
Al38	0.71191	0.28999	0.75	Al87	0.12005	0.54505	0.75	Al136	0.62679	0.9579	0.25
Al39	0.79378	0.20804	0.75	Al88	0.11858	0.63062	0.25	Al137	0.30316	0.51449	0.25
Al40	0.7944	0.29115	0.25	Al89	0.0375	0.71183	0.25	Al138	0.36353	0.56883	0.74999
Al41	0.71182	0.37311	0.25	Al90	0.03742	0.79512	0.75	Cu1	0.29622	0.41697	0.25
Al42	0.71145	0.45683	0.75	Al91	0.11938	0.71461	0.75	Cu2	0.26191	0.5618	0.75
Al43	0.79428	0.37421	0.75	Al92	0.12076	0.79643	0.25	Cu3	0.40479	0.52154	0.24999
Al44	0.79374	0.45758	0.25	Al93	0.03778	0.87747	0.25	Cu4	0.37048	0.66636	0.74999
Al45	0.70951	0.54071	0.25	Al94	0.0385	0.96015	0.75	Mg1	0.2248	0.4589	0.75
Al46	0.70741	0.62441	0.75	Al95	0.12068	0.87882	0.75	Mg2	0.29645	0.63329	0.25
Al47	0.79231	0.54128	0.75	Al96	0.1214	0.96077	0.25	Mg3	0.37024	0.45002	0.75
Al48	0.79029	0.62507	0.25	Al97	0.2068	0.36866	0.25	Mg4	0.44186	0.62447	0.24995
Al49	0.70661	0.70759	0.25	Al98	0.29373	0.35734	0.75

**Table 10:** The refined fractional coordinates in the atomic model for the 2GPBX_113_relax zone.

2GPBX_113_relax				a = 24.47 Å, b = 25.14 Å, c = 3.98 Å				α = 90.0°, β = 90.0°, γ = 91.1°			
Atoms	x	y	z	Atoms	x	y	z	Atoms	x	y	z
Al1	0.03984	0.0424	0.25	Al51	0.62617	0.04438	0.75	Al99	0.20077	0.55277	0.25
Al2	0.0412	0.1247	0.75	Al52	0.62762	0.12331	0.25	Al100	0.19969	0.63907	0.75
Al3	0.12161	0.04256	0.75	Al53	0.54422	0.20228	0.25	Cu2	0.26356	0.56201	0.75
Al4	0.12294	0.12439	0.25	Al54	0.54433	0.28239	0.75	Mg2	0.29586	0.63456	0.25
Al5	0.04287	0.20749	0.25	Al55	0.62842	0.20264	0.75	Al101	0.20234	0.71807	0.25
Al6	0.04513	0.29079	0.75	Al56	0.62942	0.28122	0.25	Al102	0.20121	0.80112	0.75
Al7	0.12455	0.20699	0.75	Al57	0.53865	0.88517	0.25	Al103	0.28231	0.7243	0.75
Al8	0.12593	0.28968	0.25	Al58	0.54058	0.96476	0.75	Al104	0.28548	0.80455	0.25
Al9	0.04609	0.37437	0.25	Al59	0.62326	0.8858	0.75	Al105	0.38177	0.36182	0.25
Al10	0.04593	0.45794	0.75	Al60	0.62504	0.9651	0.25	Mg3	0.3715	0.45147	0.75
Al11	0.12853	0.37633	0.75	Al61	0.7107	0.04409	0.25	Al106	0.46142	0.36457	0.75
Al12	0.12916	0.46016	0.25	Al62	0.71169	0.12396	0.75	Al107	0.46774	0.44518	0.25
Al13	0.04207	0.54242	0.25	Al63	0.79424	0.04356	0.75	Cu3	0.40759	0.52459	0.25
Al14	0.03834	0.62742	0.75	Al64	0.7954	0.12427	0.25	Cu4	0.36736	0.66934	0.75
Al15	0.12309	0.54451	0.75	Al65	0.71298	0.20335	0.25	Al108	0.47018	0.53317	0.75
Al16	0.11918	0.63086	0.25	Al66	0.71383	0.28302	0.75	Mg4	0.43895	0.6287	0.25
Al17	0.03651	0.71148	0.25	Al67	0.79683	0.20486	0.75	Al109	0.36938	0.72872	0.25
Al18	0.03593	0.79503	0.75	Al68	0.79772	0.2857	0.25	Al110	0.37047	0.80645	0.75
Al19	0.11776	0.71494	0.75	Al69	0.70802	0.8853	0.25	Al111	0.45448	0.72181	0.75
Al20	0.11897	0.79705	0.25	Al70	0.70938	0.96476	0.75	Al112	0.45372	0.80482	0.25
Al21	0.03661	0.8775	0.25	Al71	0.79204	0.88112	0.75	Al113	0.3056	0.51549	0.25
Al22	0.03823	0.96011	0.75	Al72	0.79299	0.96231	0.25	Al114	0.365	0.57041	0.75
Al23	0.1188	0.8793	0.75	Al73	0.87689	0.04279	0.25	Al115	0.5454	0.44537	0.75
Al24	0.12016	0.96096	0.25	Al74	0.87822	0.12464	0.75	Mg5	0.56093	0.53848	0.25
Al25	0.20443	0.04291	0.25	Al75	0.95863	0.04249	0.75	Al116	0.6305	0.43845	0.25
Al26	0.2056	0.12361	0.75	Al76	0.95997	0.12481	0.25	Cu5	0.63252	0.49781	0.75
Al27	0.28816	0.04323	0.75	Al77	0.87968	0.20624	0.25	Al117	0.52971	0.63402	0.75
Al28	0.28914	0.12309	0.25	Al78	0.88107	0.28789	0.75	Al118	0.53213	0.72202	0.25
Al29	0.20685	0.20486	0.25	Al79	0.96161	0.20708	0.75	Cu6	0.59228	0.64261	0.25
Al30	0.2078	0.28605	0.75	Al80	0.96325	0.28969	0.25	Mg6	0.62834	0.71572	0.75
Al31	0.29046	0.20242	0.75	Al81	0.88091	0.37016	0.25	Al119	0.53843	0.80264	0.75
Al32	0.2918	0.28189	0.25	Al82	0.88212	0.45227	0.75	Al120	0.54616	0.36238	0.25
Al33	0.20216	0.88152	0.25	Al83	0.96392	0.37216	0.75	Al121	0.61808	0.80539	0.25
Al34	0.20303	0.96235	0.75	Al84	0.96336	0.45572	0.25	Al122	0.62942	0.36073	0.75
Al35	0.28606	0.88418	0.75	Al85	0.8807	0.53635	0.25	Al123	0.71759	0.44289	0.75
Al36	0.28688	0.96385	0.25	Al86	0.87676	0.62269	0.75	Mg7	0.70402	0.53262	0.25
Al37	0.37222	0.0439	0.25	Al87	0.96155	0.53978	0.75	Al124	0.79754	0.44914	0.25
Al38	0.37369	0.12281	0.75	Al88	0.95781	0.62478	0.25	Al125	0.80019	0.52814	0.75
Al39	0.45676	0.04399	0.75	Al89	0.87071	0.70703	0.25	Cu7	0.73631	0.60521	0.75
Al40	0.45838	0.12321	0.25	Al90	0.87131	0.79083	0.75	Cu8	0.70348	0.75015	0.25
Al41	0.3748	0.20211	0.25	Al91	0.95394	0.70923	0.75	Al126	0.7991	0.61445	0.25
Al42	0.37658	0.2814	0.75	Al92	0.95377	0.79282	0.25	Mg8	0.77426	0.70784	0.75
Al43	0.45927	0.20244	0.75	Al93	0.87391	0.87749	0.25	Al127	0.70574	0.80836	0.75
Al44	0.46119	0.28203	0.25	Al94	0.87529	0.96016	0.75	Al128	0.71441	0.36265	0.25
Al45	0.37045	0.88596	0.25	Al95	0.9547	0.87639	0.75	Al129	0.79296	0.79784	0.25
Al46	0.37143	0.96456	0.75	Al96	0.95697	0.9597	0.25	Al130	0.79869	0.36609	0.75
Al47	0.45552	0.88481	0.75	Al97	0.20688	0.36934	0.25	Al131	0.63487	0.59675	0.75
Al48	0.45562	0.96493	0.25	Mg1	0.22556	0.45937	0.75	Al132	0.69426	0.6517	0.25
Al49	0.54146	0.044	0.25	Al98	0.29408	0.35883	0.75				
Al50	0.54309	0.1232	0.75	Cu1	0.29636	0.41704	0.25

**Table 11:** The refined fractional coordinates in the atomic model for the 2GPBX_114_relax zone.

2GPBX_114_relax				a = 24.46 Å, b = 25.18 Å, c = 3.98 Å				α = 90.0°, β = 90.0°, γ = 91.2°			
Atoms	x	y	z	Atoms	x	y	z	Atoms	x	y	z
Al1	0.03919	0.04202	0.25	Al51	0.37158	0.96499	0.75	Al101	0.87152	0.70754	0.25
Al2	0.04092	0.12499	0.75	Al52	0.45575	0.8866	0.75	Al102	0.87096	0.79067	0.75
Al3	0.1213	0.04265	0.75	Al53	0.45681	0.96518	0.25	Al103	0.95451	0.70928	0.75
Al4	0.12271	0.12485	0.25	Al54	0.54288	0.04436	0.25	Al104	0.95353	0.79231	0.25
Al5	0.04277	0.20867	0.25	Al55	0.54434	0.12303	0.75	Al105	0.87198	0.87426	0.25
Al6	0.04512	0.29243	0.75	Al56	0.62773	0.04461	0.75	Al106	0.87433	0.95802	0.75
Al7	0.12444	0.20813	0.75	Al57	0.62899	0.12383	0.25	Al107	0.95401	0.87531	0.75
Al8	0.12636	0.29108	0.25	Al58	0.54553	0.20172	0.25	Al108	0.95597	0.95831	0.25
Al9	0.04615	0.376	0.25	Al59	0.54595	0.28035	0.75	Al109	0.29497	0.35871	0.75
Al10	0.04562	0.45912	0.75	Al60	0.63016	0.20306	0.75	Al110	0.28308	0.72457	0.75
Al11	0.12889	0.37747	0.75	Al61	0.63112	0.28238	0.25	Al111	0.28617	0.80454	0.25
Al12	0.12936	0.46098	0.25	Al62	0.54054	0.88596	0.25	Al112	0.3804	0.35976	0.25
Al13	0.04071	0.54315	0.25	Al63	0.54159	0.96562	0.75	Al113	0.46199	0.36148	0.75
Al14	0.03836	0.62754	0.75	Al64	0.62473	0.88666	0.75	Al114	0.46551	0.43943	0.25
Al15	0.12156	0.5453	0.75	Al65	0.62662	0.96562	0.25	Al115	0.36806	0.72958	0.25
Al16	0.11923	0.63049	0.25	Al66	0.71175	0.04324	0.25	Al116	0.37003	0.80858	0.75
Al17	0.03675	0.71133	0.25	Al67	0.71295	0.12411	0.75	Al117	0.45157	0.72729	0.75
Al18	0.03612	0.79446	0.75	Al68	0.79436	0.04185	0.75	Al118	0.45513	0.80524	0.25
Al19	0.1183	0.71432	0.75	Al69	0.79578	0.12407	0.25	Al119	0.30445	0.51554	0.25
Al20	0.11897	0.79622	0.25	Al70	0.71424	0.20465	0.25	Al120	0.36668	0.5688	0.75
Al21	0.0365	0.87676	0.25	Al71	0.71522	0.28562	0.75	Al121	0.54903	0.43715	0.75
Al22	0.03772	0.95925	0.75	Al72	0.79713	0.20612	0.75	Al122	0.53671	0.80695	0.75
Al23	0.11875	0.87865	0.75	Al73	0.79837	0.28806	0.25	Al123	0.54708	0.35814	0.25
Al24	0.11996	0.96061	0.25	Al74	0.79814	0.3705	0.75	Al124	0.63401	0.44216	0.25
Al25	0.20414	0.04263	0.25	Al75	0.79882	0.45239	0.25	Al125	0.71481	0.44882	0.75
Al26	0.20534	0.1235	0.75	Al76	0.71709	0.61359	0.75	Al126	0.7166	0.52811	0.25
Al27	0.28809	0.0429	0.75	Al77	0.79788	0.53621	0.75	Al127	0.62213	0.808	0.25
Al28	0.28937	0.12212	0.25	Al78	0.79554	0.62139	0.25	Al128	0.63094	0.3622	0.75
Al29	0.20672	0.20454	0.25	Al79	0.78778	0.70569	0.75	Al129	0.70834	0.79742	0.75
Al30	0.20859	0.28641	0.75	Al80	0.78822	0.7892	0.25	Al130	0.71541	0.36598	0.25
Al31	0.29048	0.20109	0.75	Al81	0.70852	0.88028	0.25	Al131	0.55041	0.59792	0.25
Al32	0.29238	0.28005	0.25	Al82	0.71038	0.96218	0.75	Al132	0.61264	0.65117	0.75
Al33	0.20876	0.36927	0.25	Al83	0.79074	0.8756	0.75	Cu1	0.29795	0.41863	0.25
Al34	0.20001	0.55312	0.25	Al84	0.79266	0.95857	0.25	Cu2	0.26395	0.56308	0.75
Al35	0.2005	0.63861	0.75	Al85	0.87615	0.04172	0.25	Cu3	0.40643	0.51991	0.25
Al36	0.2023	0.71792	0.25	Al86	0.87789	0.12469	0.75	Cu4	0.3667	0.66761	0.75
Al37	0.20169	0.80076	0.75	Al87	0.95768	0.04173	0.75	Cu5	0.55034	0.49912	0.25
Al38	0.2019	0.88113	0.25	Al88	0.9594	0.12497	0.25	Cu6	0.51065	0.64682	0.75
Al39	0.20284	0.96209	0.75	Al89	0.87936	0.20745	0.25	Cu7	0.65314	0.60363	0.25
Al40	0.28599	0.88436	0.75	Al90	0.88062	0.28994	0.75	Cu8	0.61912	0.74808	0.75
Al41	0.28696	0.96368	0.25	Al91	0.96112	0.20838	0.75	Mg1	0.22678	0.46005	0.75
Al42	0.37277	0.04368	0.25	Al92	0.96309	0.29138	0.25	Mg2	0.29582	0.63389	0.25
Al43	0.37423	0.12235	0.75	Al93	0.881	0.37223	0.25	Mg3	0.37253	0.45097	0.75
Al44	0.45774	0.04412	0.75	Al94	0.88038	0.45534	0.75	Mg4	0.43458	0.63038	0.25
Al45	0.45938	0.12262	0.25	Al95	0.96357	0.37437	0.75	Mg5	0.4825	0.53639	0.75
Al46	0.37552	0.2011	0.25	Al96	0.96261	0.4574	0.25	Mg6	0.54457	0.71574	0.25
Al47	0.37657	0.28076	0.75	Al97	0.87875	0.53915	0.25	Mg7	0.62129	0.53282	0.75
Al48	0.46029	0.20155	0.75	Al98	0.8764	0.6235	0.75	Mg8	0.69033	0.70664	0.25
Al49	0.46137	0.28012	0.25	Al99	0.96009	0.54122	0.75				
Al50	0.37118	0.88636	0.25	Al100	0.95702	0.62545	0.25

**Table 12:** The refined fractional coordinates in the atomic model for the S-I phase.

S-I				a = 18.85 Å, b = 4.05 Å, c = 9.06 Å				α = 90.0°, β = 90.0°, γ = 90.0°			
Atoms	x	y	z	Atoms	x	y	z	Atoms	x	y	z
Al1	0	0	0.99983	Al15	0.19023	0	0.21569	Al29	0.33125	0.5	0.62921
Al2	0	0.5	0.49983	Al16	0.80977	0	0.78388	Al30	0.66875	0.5	0.37033
Al3	0.04719	0	0.30343	Al17	0.19023	0.5	0.71569	Cu1	0.4049	0.5	0.21593
Al4	0.95282	0	0.69619	Al18	0.80977	0.5	0.28388	Cu2	0.59509	0.5	0.78362
Al5	0.04719	0.5	0.80343	Al19	0.14163	0.5	0.41078	Cu3	0.4049	0	0.71592
Al6	0.95282	0.5	0.19619	Al20	0.85837	0.5	0.58881	Cu4	0.59509	0	0.28362
Al7	0.23839	0.5	0.01908	Al21	0.14163	0	0.91078	Mg1	0.40281	0	0.4269
Al8	0.76161	0.5	0.9805	Al22	0.85837	0	0.08881	Mg2	0.59718	0	0.57263
Al9	0.23839	0	0.51908	Al23	0.28507	0.5	0.33002	Mg3	0.40281	0.5	0.9269
Al10	0.76161	0	0.4805	Al24	0.71492	0.5	0.66954	Mg4	0.59718	0.5	0.07263
Al11	0.09453	0.5	0.10676	Al25	0.28507	0	0.83002	Al31	0.48019	0	0.14309
Al12	0.90548	0.5	0.89285	Al26	0.71492	0	0.16954	Al32	0.51981	0	0.85643
Al13	0.09453	0	0.60676	Al27	0.33125	0	0.1292	Al33	0.48019	0.5	0.64309
Al14	0.90548	0	0.39285	Al28	0.66875	0	0.87033	Al34	0.51981	0.5	0.35643
Al1	0	0	0.99983	Al15	0.19023	0	0.21569	Al29	0.33125	0.5	0.62921
Al2	0	0.5	0.49983	Al16	0.80977	0	0.78388	Al30	0.66875	0.5	0.37033
Al3	0.04719	0	0.30343	Al17	0.19023	0.5	0.71569	Cu1	0.4049	0.5	0.21593
Al4	0.95282	0	0.69619	Al18	0.80977	0.5	0.28388	Cu2	0.59509	0.5	0.78362
Al5	0.04719	0.5	0.80343	Al19	0.14163	0.5	0.41078	Cu3	0.4049	0	0.71592
Al6	0.95282	0.5	0.19619	Al20	0.85837	0.5	0.58881	Cu4	0.59509	0	0.28362
Al7	0.23839	0.5	0.01908	Al21	0.14163	0	0.91078	Mg1	0.40281	0	0.4269
Al8	0.76161	0.5	0.9805	Al22	0.85837	0	0.08881	Mg2	0.59718	0	0.57263
Al9	0.23839	0	0.51908	Al23	0.28507	0.5	0.33002	Mg3	0.40281	0.5	0.9269
Al10	0.76161	0	0.4805	Al24	0.71492	0.5	0.66954	Mg4	0.59718	0.5	0.07263
Al11	0.09453	0.5	0.10676	Al25	0.28507	0	0.83002	Al31	0.48019	0	0.14309
Al12	0.90548	0.5	0.89285	Al26	0.71492	0	0.16954	Al32	0.51981	0	0.85643
Al13	0.09453	0	0.60676	Al27	0.33125	0	0.1292	Al33	0.48019	0.5	0.64309
Al14	0.90548	0	0.39285	Al28	0.66875	0	0.87033	Al34	0.51981	0.5	0.35643

**Table 13:** The refined fractional coordinates in the atomic model for the S-II phase.

S-II				a = 19.21 Å, b = 4.05 Å, c = 16.70 Å				α = 90.0°, β = 94.4°, γ = 90.0°			
Atoms	x	y	z	Atoms	x	y	z	Atoms	x	y	z
Al1	0	0	0	Al26	0.97135	0.5	0.11495	Mg5	0.26266	0	0.44095
Al2	0	0.5	0.5	Al27	0.19387	0.5	0.18178	Mg6	0.73734	0	0.55905
Al3	0.11264	0.5	0.03542	Al28	0.80613	0.5	0.81822	Mg7	0.26266	0.5	0.94095
Al4	0.88736	0.5	0.96458	Al29	0.19387	0	0.68178	Mg8	0.73734	0.5	0.05905
Al5	0.11264	0	0.53542	Al30	0.80613	0	0.31822	Cu5	0.34888	0	0.01608
Al6	0.88736	0	0.46458	Al31	0.1571	0	0.30071	Cu6	0.65112	0	0.98392
Al7	0.22605	0	0.07248	Al32	0.8429	0	0.69929	Cu7	0.34888	0.5	0.51608
Al8	0.77395	0	0.92752	Al33	0.1571	0.5	0.80071	Cu8	0.65112	0.5	0.48392
Al9	0.22605	0.5	0.57248	Al34	0.8429	0.5	0.19929	Al43	0.42612	0	0.47551
Al10	0.77395	0.5	0.42752	Al35	0.13619	0.5	0.42135	Al44	0.57388	0	0.52449
Al11	0.33674	0.5	0.10651	Al36	0.86381	0.5	0.57865	Al45	0.42612	0.5	0.97551
Al12	0.66326	0.5	0.89349	Al37	0.13619	0	0.92135	Al46	0.57388	0.5	0.02449
Al13	0.33674	0	0.60651	Al38	0.86381	0	0.07865	Cu9	0.44025	0	0.32664
Al14	0.66326	0	0.39349	Cu1	0.23794	0.5	0.32789	Cu10	0.55975	0	0.67336
Al15	0.0812	0	0.1513	Cu2	0.76206	0.5	0.67211	Cu11	0.44025	0.5	0.82664
Al16	0.9188	0	0.8487	Cu3	0.23794	0	0.82789	Cu12	0.55975	0.5	0.17336
Al17	0.0812	0.5	0.6513	Cu4	0.76206	0	0.17211	Mg9	0.47095	0	0.10642
Al18	0.9188	0.5	0.3487	Al39	0.36817	0.5	0.36824	Mg10	0.52905	0	0.89358
Al19	0.05171	0.5	0.2675	Al40	0.63183	0.5	0.63176	Mg11	0.47095	0.5	0.60642
Al20	0.94829	0.5	0.7325	Al41	0.36817	0	0.86824	Mg12	0.52905	0.5	0.39358
Al21	0.05171	0	0.7675	Al42	0.63183	0	0.13176	Al47	0.44198	0.5	0.2336
Al22	0.94829	0	0.2325	Mg1	0.31008	0	0.24296	Al48	0.55802	0.5	0.7664
Al23	0.02865	0	0.38505	Mg2	0.68992	0	0.75704	Al49	0.44198	0	0.7336
Al24	0.97135	0	0.61495	Mg3	0.31008	0.5	0.74296	Al50	0.55802	0	0.2664
Al25	0.02865	0.5	0.88505	Mg4	0.68992	0.5	0.25704				

Tables 2 and 3 represent the models of the GPB^1^ zone with Cu or Al interstitial, respectively. Tables 4 and 5 show models containing a single GPB^2^ zone or a pair of GPB^2^ zones, respectively. Table 6 exhibits the model of the newly observed GPBX zone. Tables 7 and 8 show models of two individual GPBX zones orientated along <114>_Al_ or <113>_Al_ directions, respectively. The illustration can be found in reference [1]. Tables 9, 10 and 11 display the relaxed models of several GPBX zones listed in Tables 6, 7 and 8, respectively. Tables 12 and 13 show the models of S-I and S-II phases, respectively. Note that the corresponding raw atomic models of all precipitates listed in Tables 2 to 13 can be found in the supplementary materials as cif files.

## 2 Experimental Design, Materials and Methods

### 2.1 Materials

The investigated Al-Mg-Cu alloy (3.04% Mg, 1.01% Cu, 0.01% Si, 0.01% Fe, and Al of the remaining amount, all by mass) was provided by Furukawa sky (now, UACJ Corporation). In the factory, materials were mold cast into 400 × 170 × 40 mm bulk materials and homogenized at 773 K for 10 h. After the facing work, the billets were rolled into 3 mm thick sheets at 683 K. Finally, the sheets were cold rolled to a thickness of 1.2 mm.

The received materials were cut to small sheet-shaped samples of dimension 10 × 10 × 1.2 (in mm), and then solution heat treated (SHT) in a salt bath at 793 K for 1 h, followed by quenching in iced water (273 K). After SHT, cold rolling (CR) was performed at room temperature (approximately 293 K) with a reduction rate of 7%. The change in thickness of the sheet was monitored to determine the reduction rate. Isothermal artificial aging was performed on the A.Q. and A.R. samples in an oil bath at 443 K for 20 min and 8 h, respectively. The as-quenched and as-rolled conditions are abbreviated as “A.Q.” and “A.R.” respectively.

### 2.2 Transmission electron microscopy

For the preparation of TEM specimens, 1.2 mm thick sheet samples were first ground down to around 100 μm thickness, and then from 3 mm diameter discs were punched out of ground thin sheets. Subsequently, a Struers TenuPol-5 machine was used to electropolishing the discs until perforation. Two liters of electrolyte was prepared by mixing the 667 ml nitric acid (60.0% concentration) and 1333 ml methanol (99.0% concentration), and the temperature was kept between 243 K and 253 K during electropolishing. To reduce the amount of contamination, prior to the HAADF-STEM observations, all specimens were plasma cleaned for 3 min in a Fischione 1020 Plasma Cleaner. All the high-resolution HAADF-STEM images in this article were taken in a <100>_Al_ orientation, as precipitates extending along this direction. The instrument was a double Cs corrected JEOL ARM 200F operated at 200 kV. The convergence semi-angle was set to 28 mrad and the inner collection angle of the HAADF detector was 48 mrad.

Some of the HAADF-STEM images were filtered using a circular bandpass mask applied on the respective fast Fourier transform (FFT) to further improve the clarity, and an inverse FFT (IFFT) was performed on the masked area. This operation can cut all spatial frequencies that correspond to features in the real space smaller than 0.15 nm. The operation was performed in the software “GMS 3”.

### 2.3 First-principles calculations

The first principles calculations were carried out with density functional theory as implemented in the Vienna ab initio simulation package (VASP) [6,7]. All formation enthalpies were calculated at zero Kelvin using the projector augmented wave method within the PBE (Perdew–Burke–Ernzerhof) generalized gradient approximation [4] with a plane-wave energy cut-off of 400 eV. A Monkhorst-Pack gamma-centered k-point mesh was used, with maximal k-point distances of 0.18 Å^−1^ in each direction [5]. Partial occupancies were smeared using the first-order Methfessel-Paxton method with a smearing factor (SIGMA) of 0.2. For final energies, the tetrahedron method with Blöchl correction for the smearing [8]. The convergence criteria for the electronic convergence in the self-consistent cycles was 10^−6^ eV and the atomic positions were relaxed to a maximal atomic force of 1 meV/Å.

The zero Kelvin formation enthalpies were calculated as described by Marioara et al. [9] using a fixed aluminum lattice parameter of 4.0400 Å, corresponding to the lattice parameter relaxed with VASP using the above parameters. Not relaxing the supercell size makes it easier to compare different calculations but also overestimates the strain contribution to the formation enthalpy (corresponding to the surrounding Al being infinitely hard). A lower limit to the strain contribution was found for the structures with the largest internal pressure as listed in Table 1 by fully relaxing the supercell size (corresponding to the surrounding Al being infinitely soft). To reduce systematic errors arising from the k-point meshing of different supercell sizes, separate reference calculations for a single solute atom in the Al lattice were performed for each supercell size.

## CRediT Author Statement

**Xuanliang Chen:** Conceptualization, Investigation, Data curation, Writing - original draft, Visualization.

**Calin D. Marioara:** Formal analysis, Writing - review & editing, Supervision.

**Sigmund J. Andersen:** Methodology, Atomic models, Formal analysis, Writing - review & editing.

**Jesper Friis:** Software, Calculations of models, Writing - review & editing.

**Adrian Lervik:** Investigation.

**Randi Holmestad:** Resources, Writing - review & editing, Supervision.

**Equo Kobayashi:** Resources, Writing - review & editing, Supervision.

## Declaration of Competing Interest

The authors declare that they have no known competing financial interests or personal relationships which have or could be perceived to have influenced the work reported in this article.
